# Effect of admission in the stroke care unit versus intensive care unit on in-hospital mortality in patients with acute ischemic stroke

**DOI:** 10.1186/s12883-023-03454-6

**Published:** 2023-11-13

**Authors:** Masato Kanda, Takanori Sato, Yoichi Yoshida, Hiroyo Kuwabara, Yoshio Kobayashi, Takahiro Inoue

**Affiliations:** 1 Department of Cardiovascular Medicine, Chiba University Graduate School of Medicine, Chiba, Japan; 2 Healthcare Management Research Center, Chiba University Hospital, Chiba, Japan; 3 Department of Neurological Surgery, Chiba University Graduate School of Medicine, Chiba, Japan

**Keywords:** Acute ischemic stroke, Stroke care unit, Intensive care unit, In-hospital mortality, Length of stay, Expense

## Abstract

**Background/objective:**

Few reports have directly compared the outcomes of patients with acute ischemic stroke (AIS) who are managed in a stroke care unit (SCU) with those who are managed in an intensive care units (ICU). This large database study in Japan aimed to compare in-hospital mortality between patients with AIS admitted into SCU and those admitted into ICU.

**Methods:**

Patients with AIS who were admitted between April 1, 2014, and March 31, 2019, were selected from the administrative database and divided into the SCU and ICU groups. We calculated the propensity score to match groups for which the admission unit assignment was independent of confounding factors, including the modified Rankin scale (mRS) score. The primary outcome was in-hospital mortality, and secondary outcomes were the mRS score at discharge, length of stay (LOS), and total hospitalization cost.

**Results:**

Overall, 8,683 patients were included, and 960 pairs were matched. After matching, the in-hospital mortality rates of the SCU and ICU groups were not significantly different (5.9% vs. 7.9%, *P* = 0.106). LOS was significantly shorter (SCU = 20.9 vs. ICU = 26.2 days, *P* < 0.001) and expenses were significantly lower in the SCU group than in the ICU group (SCU = 1,686,588 vs. ICU = 1,998,260 yen, *P* < 0.001). mRS scores (score of 1–3 or 4–6) at discharge were not significantly different after matching. Stratified analysis showed that the in-hospital mortality rate was lower in the ICU group than in the SCU group among patients who underwent thrombectomy.

**Conclusions:**

In-hospital mortality was not significantly different between the ICU and SCU groups, with significantly lower costs and shorter LOS in the SCU group than in the ICU group.

**Supplementary Information:**

The online version contains supplementary material available at 10.1186/s12883-023-03454-6.

## Introduction

In a stroke unit (SU) [1], multidisciplinary teams are effective for reducing mortality, preventing the worsening or recurrence of stroke and infections, such as pneumonia, increasing the return-to-home rate, and decreasing the length of stay (LOS) [2–7]. Additionally, patients have improved long-term physical scores (such as quality of life or activities of daily living [ADL]) [8, 9]. In Japan, the stroke care unit (SCU) is equivalent to the SU defined by the European Stroke Organization, and all staff consists of neurospecific experts, however, staff of general ICU specialized in critical care [10, 11], but are not necessarily neurospecific experts. The SCU is staffed with one nurse for every three patients, which is a slightly lower nurse-to-patient ratio than that in ICUs in Japan, which are staffed with one nurse for every two patients.

Large regional variations in the SCU establishment exist [12], and several prefectures in Japan, as well as countries in which an efficient SU system is not established, may use the general ICU for treatment of patients with acute ischemic stroke (AIS). Patients with severe stroke and associated unstable vital conditions may benefit from intensive care admission [13–16]. However, treatment of AIS in the ICU places substantial burden on economic and personal resources, which may not be necessary for all patients with AIS [17–19]. There are only few reports showing direct comparisons of the outcomes of patients with AIS who are managed in SUs with those who are managed in general ICUs [20]. Therefore, this large database study in Japan aimed to compare in-hospital mortality between patients with AIS admitted to SCU and those admitted to ICU.

## Methods

### Data source

This retrospective study was approved by the ethics committee of Chiba University Hospital (approval number 3309). The study was performed in accordance with the 1975 Declaration of Helsinki. The requirement for informed consent was waived because of the anonymity of the data. This study used the Diagnosis Procedure Combination (DPC) database, and data were obtained from hospitals that were included in the DPC system and volunteered to participate in the study [21]. The DPC database organizes administrative information obtained during acute-phase hospitalization and is used for reimbursement in the per-diem payment system. The database contains patient information on demographics (e.g., age, sex, height, and weight), the most resource-consuming disease, in-hospital death, other major diagnoses and comorbidities, consciousness level, and ADL status. It also includes prescribed medications, treatment procedures, and other hospital-related information. Authors MK and TI have full access to all the data in the study and take responsibility for its integrity and data analysis.

### Data availability

Data are available to researchers on request for the purpose of reproducing the results or replicating the procedure by directly contacting the corresponding author.

### Selection of the study population

**Fig. 1:**
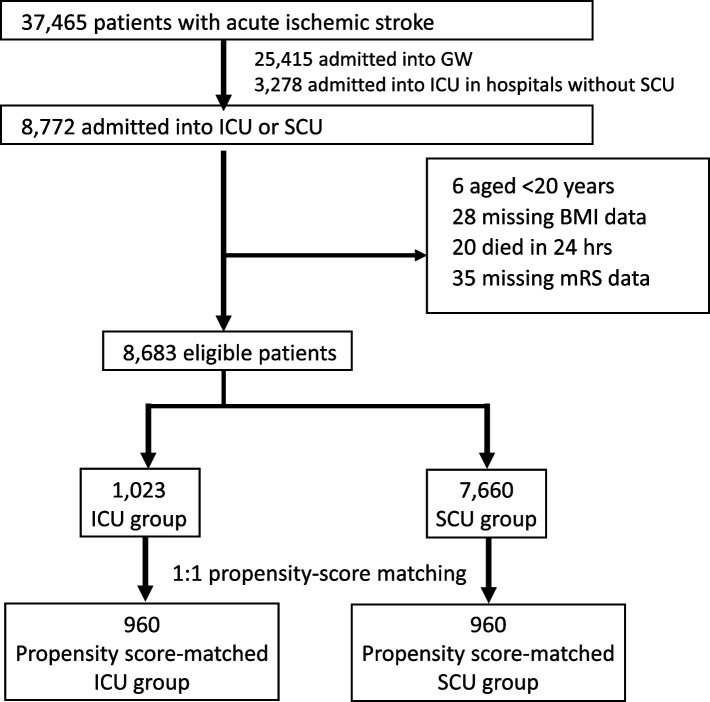
Sample selection process

A flowchart of the sample selection procedure is shown in Fig. 1. We selected patients aged ≥ 20 years with emergent AIS admission by identifying patients with the most resource-consuming disease (AIS DPC code: 0140 or 010060), based on the International Classification of Diseases (ICD-10) coding. From the 37,465 patients admitted to the 157 participating hospitals between April 1, 2014 and March 31, 2019, we excluded 25,415 patients who were admitted to general wards and 3,278 patients who were admitted to ICUs in hospitals without SCU. We identified 8,772 patients who were admitted to either the ICU or the SCU in 27 hospitals where both the ICU and SCU were available, as identified by the ICU and SCU revenue center codes. We excluded patients with missing body mass indices and modified Rankin scale (mRS) scores, and those who died within 24 h.

### Outcomes

The primary outcome was in-hospital mortality. The secondary outcomes were mRS score at discharge [22], total LOS (days from admission to discharge), and total hospitalization cost based on reimbursement of treatment costs from the DPC system.

### Exposure and baseline variables

The exposure variables were ICU or SCU admission upon hospital admission, defined by the presence of an ICU or SCU revenue center code in the administrative DPC data.

We extracted the following baseline characteristics: age, sex, body mass index, ambulance use, weekend admission, history of cerebrovascular disease, total dependence on ADL, mRS score on admission, severe impairment in consciousness (measured with the Japan Coma Scale score ≥ 20 points) on admission, AIS type (cardioembolic infarction or other), on-admission treatment (i.e., tissue plasminogen activator [tPA], percutaneous thrombectomy, percutaneous catheter intervention, or cathethrombolysis), medical history (e.g., hypertension, diabetes mellitus, dyslipidemia, ischemic heart disease, atrial fibrillation [AFib], pneumonia, asthma or chronic obstructive pulmonary disease, chronic renal disease, anemia, or cancer), and hospital admission volume of patients with AIS. The Japan Coma Scale has four categories: alert, one-, two- and three-digit codes, with each digit code having three subcategories (1, 2, and 3 in the one-digit code; 10, 20, and 30 in the two-digit code; and 100, 200, and 300 in the three-digit code) [23]. A Japan Coma Scale score of 20 points is the consciousness level (the patient opens the eye only to a loud voice or when the body is shaken). We calculated the average number of annual AIS admissions for each hospital (annual hospital volume, case/year), and the hospitals were divided into quartiles based on the number of admissions [24].

### Post-admission treatments

The following on-admission treatments were also recorded: medications (e.g., ozagrel, edaravone, argatroban, and inotropic agents), treatments (e.g., hemodiafiltration, mechanical ventilation, cardiopulmonary resuscitation, and blood transfusion), and post-admission surgery (e.g., craniotomy and endarterectomy).

### Statistical analyses

Formatting and preprocessing of the original DPC data were conducted using Python version 2.7.15 (Van Rossum G, Drake Jr FL. Python Reference Manual. Centrum voor wiskunde en informatica Amsterdam, The Netherlands). All the statistical analyses including Pearson’s chi-squared test, Welch’s t test, Student’s t test, the Mann‒Whitney U test, and propensity score analysis, were conducted using R version 3.6.2 (R Foundation for Statistical Computing, Vienna, Austria).

#### Propensity score analysis

We performed propensity score matching because nonrandom assignment to either an ICU or an SCU was likely to produce selection bias [25, 26]. The propensity score for each patient was the conditional probability of ICU admission, estimated using a logistic regression model evaluating all measured baseline variables, including on-admission treatment, as independent variables. Each patient in the ICU entry group was matched with one patient in the SCU entry group with the closest estimated propensity score within a caliper (≤ 0.20 of the pooled standard deviation of estimated logits) based on the nearest-neighbor method without replacement. For the comparison of baseline characteristics, absolute values > 10% of standardized differences were considered to indicate a significant imbalance.

Treatments after admission, in-hospital mortality, LOS, and expenses before and after matching were compared using Pearson's chi-squared test, Welch’s t test, Student’s t test, and the Mann‒Whitney U test, as appropriate. Statistical significance was set at P < 0.05.

#### Sensitivity analyses

A series of sensitivity analyses were conducted. First, we performed propensity score matching analyses among patients with 1) an mRS score of 0–3 points before the stroke, 2) an mRS score of 4–6 points before the stroke, 3) severe impairment in consciousness (JCS ≥ 20), 4) total dependence in ADL at admission (defined by the Barthel Index of zero), and 5) mechanical ventilation during admission. Second univariate linear regression analysis was performed for all (unmatched) patients using in-hospital mortality as the dependent variable and all baseline variables, including ICU admission, were selected as the independent variables. Subsequently, statistically significant variables were included in a multivariate logistic analysis.

#### Stratified analyses

We performed stratified group analyses of factors associated with stroke prognosis for interactions with ICU admission and mortality. Age (stratified at 80 years) [27], sex, stroke type, admission treatment, and comorbidity were included in the analyses. The main group was divided into cardioembolic origin and non-cardioembolic origin according to stroke type. For strokes with non-cardioembolic origin, we also described in-hospital mortality due to atherothrombotic infarction and lacuna infarction in the ICU or SCU group. In each subgroup, we created propensity scores for the conditional probability of ICU admission on day 1, estimated using a logistic regression model with a stratified group variable as an independent variable adjusted for all measured baseline variables. After propensity score matching, the in-hospital mortality of patients in the SCU was compared with corresponding patients in the ICU using Welch’s t test, Student’s t test, or Mann‒Whitney U test, as appropriate. Second, we performed a test for interaction with ICU admission using multivariable models to identify statistically significant subgroup differences with a significant interaction [28].

## Results

### Study patients

After applying the exclusion criteria, 8,683 patients were eligible (Fig. 1). Of these patients, 1,023 (11.8%) were admitted to the ICU on day 1, and 7,660 (88.2%) were admitted to the SCU. Propensity score matching created 960 pairs.

### Baseline characteristics

The baseline characteristics are shown in Table 1. In the pre-matched cohort, the ICU group had significantly more patients who arrived by ambulance; who had total dependence in ADL, severe impairment in consciousness, cardioembolic infarction, atrial fibrillation (AFib), and pneumonia; with mRS score of 0–1 or 0–2 points; who received tPA treatment; and who underwent percutaneous catheter intervention or thrombectomy. The percentage of the annual AIS admission volume in each quartile of hospital admissions differed. After matching the cohorts, the baseline characteristics, including the mRS score before stroke, were balanced.

**Table 1:** Baseline characteristics of the pre-match and matched samples

Variable	Before propensity score matching			After propensity score matching		
	**SCU** **(*n* = 7,660)**	**ICU** **(*n* = 1,023)**	**Absolute standardized difference, %**	**SCU** **(*n* = 960)**	**ICU** **(*n* = 960)**	**Absolute standardized difference, %**
**Age (years), mean (SD)**	74.9 (12.7)	75.3 (13.0)	2.8	74.6 (13.1)	75.3 (13.1)	5.6
**Male**	4,816 (58.3%)	589 (57.6%)	1.5	570 (59.4%)	552 (57.5%)	3.8
**BMI (kg/m**^**2**^**), mean (SD)**	22.8 (4.7)	22.5 (4.0)	6.5	22.6 (3.9)	22.5 (4.0)	0.9
**Ambulance use**	4,553 (59.4%)	908 (88.6/%)	71.0	859 (89.4%)	846 (88.1%)	4.3
**Weekend admission**	1,965 (25.7%)	287 (28.1%)	5.4	267 (27.8%)	267 (27.8%)	0.0
**History of cerebrovascular disease**	2,112 (27.6%)	257 (25.1%)	4.8	253 (26.3%)	242 (25.2%)	2.2
**Total dependence in ADL at admission**	1,305 (17.0%))	437 (42.7%)	58.4	364 (37.9%)	384 (40.0)	4.3
**mRS before stroke (median)**	1	0	38.5	0	0	9.4
**mRS ≤ 1**	2,751 (35.9%)	783 (76.5%)	25.1	752 (78.3%)	724 (75.4%)	6.9
**mRS ≤ 2**	4,305 (56.2%)	853 (83.4%)	12.7	808 (84.1%)	793 (82.6%)	4.2
**mRS ≤ 3**	5,394 (70.4%)	919 (89.8%)	4.2	866 (90.2%)	856 (89.2%)	3.4
**On-admission treatment**						
**TPA**	622 (8.1%)	351 (34.3%)	67.6	274 (28.5%)	296 (30.8%)	5.0
**PCI**	24 (0.3%)	21 (2.1%)	16.1	12 (1.3%)	13 (1.4%)	0.9
**Thromboprophylaxis**	9 (0.1%)	7 (0.7%)	9.0	6 (0.6%)	4 (0.4%)	2.9
**Thrombectomy**	363 (4.7%)	212 (20.1%)	49.4	175 (18.2%)	179 (18.6%)	1.1
	885 (10.2%)	885 (10.2%)				
** Severe Impairment in consciousness (JCS > 19)**	458 (6.0%)	223 (21.8%)	47.0	175 (18.2%)	191 (19.9%)	4.2
** Cardioembolic infarction**	2,064 (27.0%)	554 (54.2%)	57.7	488 (50.8%)	504 (52.5%)	3.3
**Comorbidity**						
**Hypertension**	3,554 (46.4%)	464 (45.3%)	2.1	428 (44.6%)	431 (44.9%)	0.6
**Diabetes**	1,641 (21.4%)	199 (19.5%)	4.9	211 (22.0%)	188 (19.6%)	5.9
**Dyslipidemia**	1,948 (25.4%)	235 (23.0%)	5.7	250 (26.0%)	228 (23.8%)	5.3
**Ischemic heart disease**	354 (4.6%)	37 (3.6%)	5.1	34 (3.5%)	36 (3.8%)	1.1
**Atrial fibrillation**	1417 (18.5%)	353 (34.5%)	36.9	320 (33.3%)	321 (33.4%)	0.2
**Pneumonia**	190 (2.5%)	44 (4.3%)	10.1	30 (3.1%)	42 (4.4%)	6.6
**COPD or asthma**	94 (1.2%)	10 (1.0%)	2.4	6 (0.6%)	10 (1.0%)	4.6
**CRD**	302 (3.9%)	23 (2.2%)	9.8	25 (2.6%)	20 (2.1%)	3.4
**Anemia**	103 (1.3%)	13 (1.3%)	0.7	13 (1.4%)	11 (1.1%)	1.9
**Cancer**	399 (5.2%)	40 (3.9%)	6.2	44 (4.6%)	39 (4.1%)	2.6
Annual hospital volume, case/year			21.7			5.7
Quartile 1 (< 21)	6 (0.1%)	2 (0.2%)		1 (0.1%)	2(0.2%)	
Quartile 2 (21–100)	63 (0.8%)	36 (3.5%)		24 (2.5%)	23 (2.4%)	
Quartile 3 (101–323)	723 (9.4%)	65 (6.4%)		47 (4.9%)	58 (6.0%)	
Quartile 4 (≥ 324)	6,868 (89.7%)	920 (89.9%)		888 (92.5%)	877 (91.4%)	

Data are shown as numbers (%) unless otherwise stated

*BMI* Body mass index, *COPD* Chronic obstructive pulmonary disease, *CRD* Chronic renal disease, *DCM* Dilated cardiomyopathy, *GW* General ward, *HF* Heart failure, *ICU* Intensive care unit, *IHD* Ischemic heart disease, *NYHA* New York Heart Association, *PH* Pulmonary hypertension, *pre-ADL* Activity of daily living at admission, *PVD* Peripheral vascular disease, *SD* Standard deviation, *VHD* Valvular heart disease

### Post-admission treatments

Table 2 shows the post-admission treatments for the pre-matched and matched samples. In the pre-matched cohort, the use of edaravone and inotropes was significantly higher and the use of ozagrel and argatroban was significantly lower in the ICU group than in the SCU group. After matching, the use of argatroban and inotropes was significantly higher and the use of ozagrel was significantly lower in the ICU group than in the SCU group. In the pre-matched sample, mechanical ventilation, cardiopulmonary resuscitation, and blood transfusion were performed more frequently, and hemodiafiltration was less frequently performed in the ICU group compared with the SCU group. In the matched cohort, mechanical ventilation and blood transfusions were performed more frequently in the ICU group compared with the SCU group.

**Table 2:** Post-admission interventions in the pre-matched and matched samples

Variable	Before propensity score matching				After propensity score matching			
	**All patients** **(*n* = 8,683)**	**SCU** **(*n* = 7,660)**	**ICU** **(*n* = 1,023)**	***P*-value**	**All patients** **(*n* = 1,920)**	**SCU** **(*n* = 960)**	**ICU** **(*n* = 960)**	***P*-value**
**Drugs**								
**ozagrel**	1,805 (20.8%)	1,729 (22.6%)	76 (7.4%)	< 0.001	205 (10.7%)	131 (13.6%)	74 (7.7%)	< 0.001
**edaravone**	6,070 (69.9%)	5,280 (68.9%)	790 (77.2%)	< 0.001	1,464 (76.3%))	731 (76.1%)	733 (76.4%)	0.915
**argatroban**	2,833 (32.6%)	2,585 (33.7%)	248 (24.2%)	< 0.001	483 (25.2%)	238 (24.8%)	245 (25.5%)	0.017
**inotrope**	867 (10.0%)	680 (8.9%)	187 (18.3%)	< 0.001	295 (15.4%)	124 (12.9%)	171(17.8%)	0.003
**Procedures**								
**hemodiafiltration**	201 (2.3%)	188 (2.5%)	13 (1.3%)	0.018	31 (1.6%)	18 (1.9%)	13 (1.4%)	0.366
**mechanical ventilation**	205 (2.4%)	115 (1.5%)	90 (8.8%)	< 0.001	103(5.4%)	27 (2.8%)	76 (7.9%)	< 0.001
**CPR**	19 (0.2%)	12 (0.2%)	7 (0.7%)	0.001	13 (0.7%)	6 (0.6%)	7 (0.7%)	0.781
**blood transfusion**	237 (2.7%)	153 (2.0%)	84 (8.2%)	< 0.001	102 (5.3%)	24 (2.5%)	78 (8.1%)	< 0.001
**craniotomy**	0 (0.0%)	0 (0.0%)	0 (0.0%)	NaN	0 (0.0%)	0 (0.0%)	0 (0.0%)	NaN
**endarterectomy**	40 (0.4%)	37 (0.5%)	3 (0.2%)	0.400	4 (0.2%)	1(0.1%)	3 (0.3%)	0.317

Data are presented as numbers (%)

*SCU* Stroke care unit, *ICU* Intensive care unit, *CPR* Cardiopulmonary resuscitation

### Comparison of outcomes

In the pre-matched cohort, the in-hospital mortality rate in the SCU group was significantly lower than that in the ICU group. After matching, the in-hospital mortality rates of the SCU and ICU groups were not significantly different (SCU = 5.9% vs. ICU = 7.9%, *P* = 0.106) (Table 3). LOS was significantly shorter (SCU = 20.9 vs. ICU = 26.2 days, P < 0.001 after matching), and expenses were significantly lower in the SCU group than in the ICU group (SCU = 1,686,588 yen vs. ICU = 1,998,260 yen, P < 0.001 after matching) (Table 3). The percentages of patients with mRS scores of 0–1, 0–2, 0–3,4,5, or 4–6 points at discharge were significantly higher in the SCU group before matching. However, the differences for all, except the difference in the rate of mRS score of 5, which was significantly higher in the SCU group than in the ICU group, were not statistically significant after matching.

**Table 3:** Outcomes in the pre-match and matched samples

Variable	Before propensity score matching				After propensity score matching			
	**SCU** **(*n* = 7,660)**	**ICU** **(*n* = 1,023)**	**OR** **(95% CI)**	***P*-value**	**SCU** **(*n* = 960)**	**ICU** **(*n* = 960)**	**OR** **(95% CI)**	***P*-value**
**In-hospital mortality**	208 (2.7)	87 (8.5)	3.33 (2.57–4.31)	< 0.001	57 (5.9)	76 (7.9)	1.36 (0.95–1.9)	0.106
**mRS ≤ 1 at discharge**	2,751 (35.9)	272 (26.6)	0.65 (0.56–0.75)	< 0.001	291 (30.3)	256 (26.6)	0.84 (0.69–1.02)	0.077
**mRS ≤ 2 at discharge**	4,305 (56.2)	426 (41.6)	0.56 (0.49–0.63)	< 0.001	435 (45.3)	403(42.0)	0.87 (0.73–1.05)	0.141
**mRS ≤ 3 at discharge**	5,394 (70.4)	573 (56.0)	0.53 (0.47–0.61)	< 0.001	562 (58.5)	543 (56.6)	0.92 (0.77–1.11)	0.381
**mRS = 4 at discharge**	1,369 (17.9)	235 (23.0)	0.73 (0.62–0.85)	< 0.001	191 (19.9)	223 (23.2)	0.82 (0.66–1.02)	0.075
**mRS = 5 at discharge**	699 (9.1)	130 (12.7)	0.69 (0.56–0.84)	< 0.001	154 (16.0)	120 (12.5)	1.34 (1.03–1.73)	0.0265
**mRS = 4, 5 or 6 at discharge**	2,266 (29.5)	450 (44.0)	0.53 (0.47–0.61)	< 0.001	397 (41.3)	417 (43.4)	0.91 (0.77–1.10)	0.3557
	**SCU** **(*n* = 7,660)**	**ICU** **(*n* = 1,023)**	**Difference in mean** **(95% CI)**	***P*-value**	**SCU** **(*n* = 960)**	**ICU** **(*n* = 960)**	**Difference in mean** **(95% CI)**	***P*-value**
**LOS (SD), days**	18.6 (15.5)	26.5 (20.7)	7.9 (6.7–9.3)	< 0.001	20.9 (15.8)	26.2 (19.1)	5.2 (3.7–6.8)	< 0.001
**Expense (SD), yen**	1,305,616 (844,992)	2,051,805 (1,266,781)	746,090 (666,105–826,074)	< 0.001	1,686,588 (1,047,943)	1,998,260 (1,250,707)	311,672 (208,387–414,956)	< 0.001

Data are presented as number (%)

*SCU* Stroke care unit, *ICU* Intensive care unit, *OR* Odds ratio, *CI* Confidence interval, *mRS* Modified Rankin Scale, *LOS* Length of stay, *SD* Standard deviation

Sensitivity analyses showed that, among patients with severe stroke, the in-hospital mortality between the two groups was not significantly different (Additional file 1). Among the patients with AIS who underwent mechanical ventilation during admission, in-hospital mortality in the ICU group was lower than that in the SCU group, but the difference was not significant (Additional file 2). Univariate logistic analysis, in which in-hospital mortality was the dependent variable as a function of independent baseline confounding factors and on-admission interventions, was performed across all (unmatched) patient groups. ICU admission was a significant factor for in-hospital mortality (Additional file 3). Further, statistically significant variables were included in a multivariate logistic analysis, and ICU admission was identified as a significant factor of in-hospital mortality.

### Stratified analyses

**Fig. 2:**
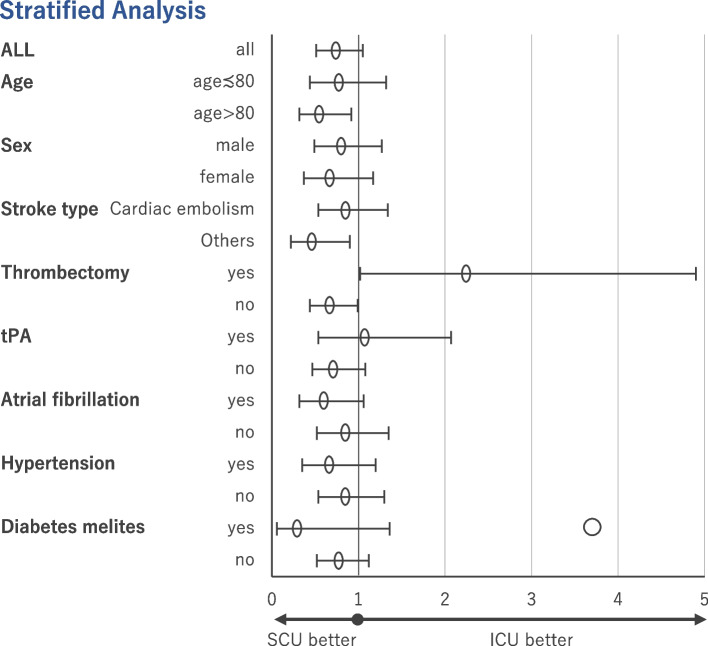
Stratified analysis of in-hospital mortality in the matched sample

We further examined the effect of baseline variables on differences in in-hospital mortality. Matching analyses in the stratified groups showed significant effect modifications by age, thrombectomy, and AFib (Fig. 2 and Additional file 4).

Among patients aged > 80 years, in-hospital mortality in the ICU group was significantly higher, whereas among patients aged ≤ 80 years, no significant difference was observed between the ICU and SCU groups. Compared with the SCU group, in-hospital mortality in the ICU group was significantly higher among patients who did not undergo thrombectomy, and was significantly lower among those who underwent thrombectomy.

## Discussion

In this retrospective cohort study, we compared in-hospital mortality between patients with AIS who were admitted into SCUs and those who were admitted into ICUs. We identified a large number of patients with AIS (8,772) who were admitted into either SCU or ICU. The 27 hospitals included in this study had both an ICU and SCU, are tertiary stroke centers and considered tertiary health facilities, and finalize necessary specialized procedures for both stroke and life support treatments. Using propensity score matching, we found no significant differences in baseline characteristics between the two groups. There was no significant difference in in-hospital mortality between the two groups, but patients in the SCU group had significantly shorter LOS and lower expense. For the mRS scores, mRS scores of 5 was significantly higher in the SCU group than in the ICU group. However, as the mRS score of 6 (which equals mortality) tended to be higher in the ICU group than in the SCU group, the number of patients with mRS scores of 4–6 was not significantly different.

In several reports, it has been recommended that severe stroke with vital sign instability (e.g., requiring mechanical intubation or cardiovascular support) is an indication for ICU admission [14, 16]. Severe AIS can cause secondary brain injuries, such as cerebral edema, hemorrhagic transformation, and progressive stroke, and it requires neurospecific critical care [13]. In this study, the sensitivity analysis showed that in patients with moderate or severe AIS (characterized by Japan Coma Scale score ≥ 20 points in consciousness or total dependence for ADL), in-hospital mortality in the SCU group was not significantly different from that in the ICU group. Univariate and multivariate analyses showed the favorable association of SCU on admission with in-hospital mortality. These results may suggest that even with slightly reduced monitoring intensity, patients with moderate to severe AIS can receive appropriate treatment in SCUs that are staffed by neurospecific experts.

In this study, the ICU group required significantly more resources than the SCU group. The difference is partly explained by the fact that ICU admission fee per day is higher than that for SCU in Japan. In addition, the LOS in the SCU group was shortened by approximately 5 days, which may also partially account for the lower expense. Generally, all the ICU-level procedures, including ventilator or ECMO procedures can be used in the SCU, if necessary. However, because of the low therapeutic threshold of ICU staff for life-saving procedures or the differences in the training level for stroke, ICU-level therapies may tend to be used more often or for longer period for patients in the ICU with the same AIS severity, which may cause the differences in cost [29]. As admission hospitals are usually selected by paramedics in Japan, triage for appropriate admission of patients with AIS may be difficult if the paramedics are not neurological experts [30]. In addition, doctors who admit the patients may have no choice but to use ICUs for admission if the hospitals do not have SCUs. Although in this study, the original number of patients in the ICU was smaller than that of patients in the SCU (1,023 vs. 7,660), we excluded 3,278 ICU admissions because they occurred in hospitals without SCU. Therefore, the total ICU admission rate of patients with AIS is comparable to that of SCU, and appropriate ICU admission is important in terms of cost-effectiveness.

Several factors may explain the favorable effects of SCU admissions. The key components of the SCU are the development of evaluation procedures (e.g., assessment of medical, nursing, and therapeutic measures), early management (e.g., early release from bed rest, avoidance of urinary catheterization, and early response to hyperglycemia, hypoxemia, and suspected infection), simultaneous rehabilitation with organized multidisciplinary collaboration, and early assessment for discharge [31]. Although it was not described in this study, care provided in the SU reduces mortality because of early and comprehensive rehabilitation interventions [32]. Patients with AIS in Japan are supposed to start rehabilitation during ICU/SCU stay with the permission of the staff in the units, and continue it in the general ward until discharge. If a disorder exists, the patients can be transferred to rehabilitation hospitals to continue rehabilitation for 2–3 months [33]. The SCU is staffed with experts, including stroke experts, who are available 24 h a day, thus, optimizing all aspects of medical therapies [34], whereas ICU staff specialize in critical care [10, 11]. The level of neurological care of ICU staff depends on each hospital. Early mobilization in the SCU may reduce stroke progression/recurrence and the occurrence of complications of immobility, pneumonia and other infections, and pressure sores, thus, leading to earlier discharge.

Stratified group analyses demonstrated significant effect modifications due to several confounding factors. In previous reports, researchers have described the controversial effects of age and sex differences on the prognosis of patients [27, 35]. In this study, no association was found between sex and the in-hospital mortality of patients with AIS admitted to the ICU or SCU. However, some association between age and in-hospital mortality was observed. Among patients aged ≥ 80 years, in-hospital mortality in the SCU group was significantly lower than that in the ICU group; whereas, among patients aged < 80 years, there was no significant difference in in-hospital mortality between the two groups. Frailty is reportedly associated with the mortality of patients with AIS [36] and strongly associated with age [37]. For elderly patients, early ambulation is important for the prevention of rapid ADL decline due to frailty. Therefore, the quality of care may be more important than that for younger patients. The type of stroke (i.e., cardioembolic or not) had no association with the admission type. Among patients with atherothrombotic infarction or lacuna infarction, in-hospital mortality was not significantly different between the ICU and SCU groups (Additional file 5). However, the admission unit was associated with AFib. Patients with AFib benefited from SCU admission, although this effect was not significant in the stratified analysis. Smaal et al. reported no interaction between AFib and thrombectomy [38]. However, frailty is more prevalent in patients with AFib [39, 40]. Therefore, the early mobilization practiced in the SCU may have beneficial effects in patients with AFib and frailty.

In a cohort of patients with AIS who underwent thrombectomy, serious AIS with sudden vital changes due to the recurrence of stroke or post-infarction hemorrhage was more common [41, 42]. Higher nurse-to-patient ratio in the ICU can enable rapid response to sudden vital changes because of close monitoring. For patients with AIS in the SCU who undergo thrombectomy, more intensive monitoring may be required to address sudden changes in their condition. The result of patients with AIS who underwent mechanical ventilation during admission again showed the need for close monitoring of patients with AIS with cardiopulmonary instability.

This present study was the first study to compare the outcomes of patients with AIS who are managed in SUs with those who are managed in general ICUs, using a large database. The result of the study proposed the additional information in the selection of admission units for patients with AIS, and provided new evidence of the need for its aggressive implementation of SUs in countries where SU is not widespread.

The present study has several limitations. First, as this study used administrative DPC data, it lacked detailed information, such as the National Institutes of Health Stroke Scale score (a widely used stroke severity assessment tool that measures the level of consciousness, eye movements, integrity of visual fields, facial movements, arm and leg muscle strength, sensation, coordination, language, speech, and neglect, and is reportedly a strong predictor of outcomes after stroke [43, 44]), vital signs, laboratory parameters, and radiographic data. Second, this observational study was not randomized: unmeasured confounding factors may have affected the relationship between ICU and SCU admissions and their outcomes. Furthermore, the precise difference in drug treatment between the two groups was untraceable, and due to the relatively long hospital stays of patients with AIS in this study, caution must be exercised when applying our findings to different cohorts of patients. Another limitation is that DPC data were related to only admission and not to follow-up. Therefore, the prognosis at 90 days or later was not traceable. Finally, our data did not account for patient transfers. Patients admitted to the ICU may be directly transferred to the general ward or SCU, and those who are admitted to the SCU may be transferred to the ICU if they need maximum intensive care (such as extracorporeal membrane oxygenation). In our study, the average stay in the ICU/SCU and general ward was not determined.

## Conclusions

This propensity score-matched observational study showed that, in general, the in-hospital mortality of patients with AIS managed in the SCUs was not significantly different from that of patients managed in ICUs, with significantly lower cost and shorter LOS in the SCU group than in the ICU group.

### Supplementary Information

**Additional file 1.** In-hospital mortality based on stroke severity in each admission category.

**Additional file 2.** In-hospital mortality in patients with AIS to whom mechanical ventilation was administered.

**Additional file 3.** Univariate and multivariate analyses of factors (unmatched) associated with in-hospital mortality.

**Additional file 4.** Stratified analysis of in-hospital mortality in the matched sample.

**Additional file 5.** In-hospital mortality based on stroke type.

## Data Availability

Data are available to researchers on request for the purpose of reproducing the results or replicating the procedure by directly contacting the corresponding author.

## References

[1] Ringelstein EB, Chamorro A, Kaste M, Langhorne P, Leys D, Lyrer P (2013). European stroke organisation recommendations to establish a stroke unit and stroke center. Stroke.

[2] Turner M, Barber M, Dodds H, Dennis M, Langhorne P, Macleod MJ (2015). The impact of stroke unit care on outcome in a Scottish stroke population, taking into account case mix and selection bias. J Neurol Neurosurg Psychiatry.

[3] Zhu HF, Newcommon NN, Cooper ME, Green TL, Seal B, Klein G (2009). Impact of a stroke unit on length of hospital stay and in-hospital case fatality. Stroke.

[4] Stroke Unit Trialists’ Collaboration (1997). How do stroke units improve patient outcomes? A collaborative systematic review of the randomized trials Stroke Unit Trialists Collaboration. Stroke.

[5] Govan L, Langhorne P, Weir CJ (2007). Does the prevention of complications explain the survival benefit of organized inpatient (stroke unit) care?: further analysis of a systematic review. Stroke.

[6] Stroke Unit Trialists’ Collaboration (1997). Collaborative systematic review of the randomised trials of organised inpatient (stroke unit) care after stroke Stroke Unit Trialists' Collaboration. BMJ.

[7] Tamm A, Siddiqui M, Shuaib A, Butcher K, Jassal R, Muratoglu M (2014). Impact of stroke care unit on patient outcomes in a community hospital. Stroke.

[8] Indredavik B, Bakke F, Slørdahl SA, Rokseth R, Håheim LL (1998). Stroke unit treatment improves long-term quality of life: a randomized controlled trial. Stroke.

[9] Indredavik B, Bakke F, Slordahl SA, Rokseth R, Hâheim LL (1999). Stroke unit treatment. 10-year follow-up. Stroke.

[10] Bray K, Wren I, Baldwin A, St Ledger U, Gibson V, Goodman S, Walsh D (2010). Standards for nurse staffing in critical care units determined by: the British association of critical care nurses, the critical care networks national nurse leads, royal college of nursing critical care and in-flight forum. Nurs Crit Care.

[11] Katz J, Powers M, Amusina O (2021). A review of procedural skills performed by advanced practice providers in emergency department and critical care settings. Dis Mon.

[12] Nilanont Y, Nidhinandana S, Suwanwela NC, Hanchaiphiboolkul S, Pimpak T, Tatsanavivat P (2014). Quality of acute ischemic stroke care in Thailand: a prospective multicenter countrywide cohort study. J Stroke Cerebrovasc Dis.

[13] Bevers MB, Kimberly WT (2017). Critical care management of acute ischemic stroke. Curr Treat Options Cardiovasc Med.

[14] Kirkman MA, Citerio G, Smith M (2014). The intensive care management of acute ischemic stroke: an overview. Intensive Care Med.

[15] Robba C, Giovannini M, Meyfroidt G, van der Jagt M, Citerio G, Smith M (2022). Intensive care admission and management of patients with acute ischemic stroke: a cross-sectional survey of the European Society of Intensive Care Medicine. J Neurosurg Anesthesiol.

[16] Smith M, Reddy U, Robba C, Sharma D, Citerio G (2019). Acute ischaemic stroke: challenges for the intensivist. Intensive Care Med.

[17] Jeng JS, Huang SJ, Tang SC, Yip PK (2008). Predictors of survival and functional outcome in acute stroke patients admitted to the stroke intensive care unit. J Neurol Sci.

[18] Sadaka F, Cytron MA, Fowler K, Javaux VM, O'Brien J (2016). A model for identifying patients who may not need neurologic intensive care unit admission: resource utilization study. J Intensive Care Med.

[19] Zimmerman JE, Kramer AA (2010). A model for identifying patients who may not need intensive care unit admission. J Crit Care.

[20] Tran QK, Yarbrough KL, Capobianco P, Chang WW, Jindal G, Medic A (2020). Comparison of outcomes after treatment of large vessel occlusion in a critical care resuscitation unit or a neurocritical care unit. Neurocrit Care.

[21] Kanda M, Tateishi K, Nakagomi A, Iwahana T, Okada S, Kuwabara H (2021). Association between early intensive care or coronary care unit admission and post-discharge performance of activities of daily living in patients with acute decompensated heart failure. PLoS One.

[22] van Swieten JC, Koudstaal PJ, Visser MC, Schouten HJ, van Gijn J (1988). Interobserver agreement for the assessment of handicap in stroke patients. Stroke.

[23] Shigematsu K, Nakano H, Watanabe Y (2013). The eye response test alone is sufficient to predict stroke outcome–reintroduction of Japan Coma Scale: a cohort study. BMJ Open.

[24] Kuwabara H, Fushimi K, Matsuda S (2011). Relationship between hospital volume and outcomes following primary percutaneous coronary intervention in patients with acute myocardial infarction. Circ J.

[25] Inoue T, Fushimi K (2013). Stroke care units versus general medical wards for acute management of stroke in Japan. Stroke.

[26] Isogai T, Matsui H, Tanaka H, Fushimi K, Yasunaga H (2016). Atrial natriuretic peptide therapy and in-hospital mortality in acute myocardial infarction patients undergoing percutaneous coronary intervention. Int J of Cardiol.

[27] Nogueira RG, Jadhav AP, Haussen DC, Bonafe A, Budzik RF, Bhuva P (2018). Thrombectomy 6 to 24 hours after stroke with a mismatch between deficit and infarct. N Engl J Med.

[28] Ferreira JC, Patino CM (2017). Subgroup analysis and interaction tests: why they are important and how to avoid common mistakes. J Bras Pneumol.

[29] Lone NI, Walsh TS (2011). Prolonged mechanical ventilation in critically ill patients: epidemiology, outcomes and modelling the potential cost consequences of establishing a regional weaning unit. Crit Care.

[30] Okuno Y, Yamagami H, Kataoka H, Tahara Y, Tonomura S, Tokunaga H (2020). Field assessment of critical stroke by emergency services for acute delivery to a comprehensive stroke center: FACE2AD. Transl Stroke Res.

[31] Langhorne P, Pollock A, Stroke Unit Trialists' Collaboration (2002). What are the components of effective stroke unit care?. Age Ageing.

[32] Indredavik B, Bakke F, Slordahl SA, Rokseth R, Hâheim LL (1999). Treatment in a combined acute and rehabilitation stroke unit: which aspects are most important. Stroke.

[33] Maeshima S OA. Community-based rehabilitation after brain infarction in Japan: from the acute phase to home. Brisbane: Exon Publications; 2021. Cerebral Ischemia. https://www.ncbi.nlm.nih.gov/books/nbk575733/10.36255/exonpublications.Cerebralischemia.2021.Rehabilitation. Accessed 17 Apr 2023. 34905308

[34] Hisaka Y, Ito H, Yasuhara Y, Takase K, Tanioka T, Locsin R (2021). Nurses' awareness and actual nursing practice situation of stroke care in acute stroke units: a Japanese cross-sectional web-based questionnaire survey. Int J Environ Res Public Health.

[35] Rexrode KM, Madsen TE, Yu AYX, Carcel C, Lichtman JH, Miller EC (2022). The impact of sex and gender on stroke. Circ Res.

[36] Tan BYQ, Ho JSY, Leow AS (2022). Effect of frailty on outcomes of endovascular treatment for acute ischaemic stroke in older patients. Age Ageing.

[37] Yang F, Li N, Yang L, Chang J, Yan A, Wei W (2022). Association of pre-stroke frailty with prognosis of elderly patients with acute cerebral infarction: a cohort study. Front Neurol.

[38] Smaal JA, de Ridder IR, Heshmatollah A, van Zwam WH, Dippel D, Majoie CB (2020). Effect of atrial fibrillation on endovascular thrombectomy for acute ischemic stroke. A meta-analysis of individual patient data from six randomised trials: Results from the HERMES collaboration. Eur Stroke J.

[39] Diemberger I, Fumagalli S, Mazzone AM, Bakhai A, Reimitz PE, Pecen L (2022). Perceived vs. objective frailty in patients with atrial fibrillation and impact on anticoagulant dosing: an ETNA-AF-Europe sub-analysis. Europace..

[40] Proietti M, Romiti GF, Raparelli V, Diemberger I, Boriani G, Dalla Vecchia LA (2022). Frailty prevalence and impact on outcomes in patients with atrial fibrillation: a systematic review and meta-analysis of 1,187,000 patients. Ageing Res Rev.

[41] Duan Y, Shammassian B, Srivatsa S, Sunshine K, Chugh A, Pace J (2022). Bypassing the intensive care unit for patients with acute ischemic stroke secondary to large-vessel occlusion. J Neurosurg.

[42] Javed K, Boyke A, Naidu I (2022). Predictors of radiographic and symptomatic hemorrhagic conversion following endovascular thrombectomy for acute ischemic stroke due to large vessel occlusion. Cureus.

[43] Brott T, Adams HP, Olinger CP, Marler JR, Barsan WG, Biller J (1989). Measurements of acute cerebral infarction: a clinical examination scale. Stroke.

[44] Kwah LK, Diong J (2014). National Institutes of Health Stroke Scale (NIHSS). J Physiother.

