# Supplementary material for: Effect of admission in the stroke care unit versus intensive care unit on in-hospital mortality in patients with acute ischemic stroke
Source: BMC Neurol. 2023 Nov 13;23:402. doi: 10.1186/s12883-023-03454-6 (PMC10641943; doi:10.1186/s12883-023-03454-6)
Supplement: Supplementary file 1 — Additional file 1. In-hospital mortality based on stroke severity in each admission category. [file 12883_2023_3454_MOESM1_ESM.docx]

**Additional file 1. In-hospital mortality based on stroke severity in each admission category**

| **Variable** | **Before propensity score matching** | | | | | **After propensity score matching** | | | | |
| --- | --- | --- | --- | --- | --- | --- | --- | --- | --- | --- |
|  | **No. of patients** | **SCU** | **ICU** | **OR (95% CI)** | **P-value** | **No. of patients** | **SCU** | **ICU** | **OR (95% CI)** | **P-value** |
| **mRS score before stroke (median)** |  |  |  |  |  |  |  |  |  |  |
| **mRS 0–3** | 6,782 vs. 919 | 152 (2.2) | 75 (8.2) | 0.26 (0.19–0.34) | <0.001 | 852 vs. 852 | 46 (5.4) | 61 (7.2) | 0.74 (0.50–1.01) | 0.162 |
| **mRS 4–5** | 878 vs. 104 | 56 (6.4) | 12 (11.5) | 0.52 (0.27–1.01) | 0.079 | 89 vs. 89 | 7 (7.9) | 10 (11.2) | 0.67 (0.24–1.86) | 0.610 |
| **Severe impairment in consciousness (JCS ≥20)** | 458 vs. 223 | 78 (17.0) | 54 (24.2) | 0.64 (0.43–0.95) | 0.034 | 171 vs. 171 | 33 (19.3) | 42 (24.6) | 0.73 (0.44–1.23) | 0.296 |
| **Total dependence for ADL at admission** | 1,305 vs. 437 | 108 (8.3) | 60 (13.7) | 0.51 (0.36–0.71) | 0.001 | 372 vs. 372 | 35 (9.4) | 46 (12.4) | 0.74 (0.46–1.17) | 0.239 |

Data are presented as numbers (rates). SCU, stroke care unit; ICU, intensive care unit; OR: odds ratio; CI, confidence interval; mRS, modified Rankin scale; JCS, Japan Coma Scale; ADL, activities of daily living

Description of data: This is a table that reports the in-hospital mortality of patients admitted in SCUs and ICUs based on stroke severity before and after propensity score matching.
