# Supplementary material for: Effect of admission in the stroke care unit versus intensive care unit on in-hospital mortality in patients with acute ischemic stroke
Source: BMC Neurol. 2023 Nov 13;23:402. doi: 10.1186/s12883-023-03454-6 (PMC10641943; doi:10.1186/s12883-023-03454-6)
Supplement: Supplementary file 2 — Additional file 2. In-hospital mortality in patients with AIS to whom mechanical ventilation was administered. [file 12883_2023_3454_MOESM2_ESM.docx]

**Additional file 2. In-hospital mortality in patients with AIS to whom mechanical ventilation was administered**

| **Variable** | **Before propensity score matching** | | | | | **After propensity score matching** | | | | |
| --- | --- | --- | --- | --- | --- | --- | --- | --- | --- | --- |
|  | **No. of patients** | **SCU** | **ICU** | **OR (95% CI)** | **P-value** | **No. of patients** | **SCU** | **ICU** | **OR (95% CI)** | **P-value** |
| Mechanical ventilation during admission | 115 vs. 90 | 32 (27.8) | 25 (27.8) | 1.00 (0.54–-1.86) | 1.000 | 46 vs. 46 | 14 (30.4) | 10 (21.7) | 1.58 (0.61–4.04) | 0.476 |

Data are presented as numbers (rates). AIS, acute ischemic stroke; SCU, stroke care unit; ICU, intensive care unit; OR: odds ratio; CI, confidence interval

Description of data: This is a table that reports the in-hospital mortality of patients admitted in SCUs and ICUs who received mechanical ventilation before and after propensity score matching.
