# Supplementary material for: Effect of admission in the stroke care unit versus intensive care unit on in-hospital mortality in patients with acute ischemic stroke
Source: BMC Neurol. 2023 Nov 13;23:402. doi: 10.1186/s12883-023-03454-6 (PMC10641943; doi:10.1186/s12883-023-03454-6)
Supplement: Supplementary file 3 — Additional file 3. Univariate and multivariate analyses of factors (unmatched) associated with in-hospital mortality. [file 12883_2023_3454_MOESM3_ESM.docx]

**Additional file 3****.** **Univariate and multivariate analyses of factors (unmatched) associated with in-hospital mortality**

|  | **Univarite logistic analysis** | | **Multivarite logistic analysis** | |
| --- | --- | --- | --- | --- |
| **Variables** | **Odds ratio (95% CI)** | **P-value** | **Odds ratio (95% CI)** | **P-value** |
| **Use of ICU on admission** | 3.49 (2.72–4.48) | <0.001 | 1.66 (1.23–2.24) | <0.001 |
| **Age >80 years** | 2.67 (2.12–3.37) | <0.001 | 1.48 (1.14–1.93) | 0.0031 |
| **Male sex** | 1.20 (0.96–1.50) | 0.1145 |  |  |
| **BMI** | 0.93 (0.91–0.97) | <0.001 | 1.00 (0.98–1.02) | 0.6937 |
| **Ambulance use** | 3.97 (2.86–5.51) | <0.001 | 1.60 (1.12–2.29) | 0.0096 |
| **Weekend admission** | 1.20 (0.94–1.54) | 0.1391 |  |  |
| **History of cerebrovascular disease** | 1.33 (1.04–1.71) | 0.0230 | 1.15 (0.87–1.52) | 0.3385 |
| **total dependence for ADL at admission** | 5.70 (4.53–7.17) | <0.001 | 2.43 (1.88–3.16) | <0.001 |
| **mRS score before stroke (median)** |  |  |  |  |
| **mRS ≤1** | 0.61 (0.49–0.76) | <0.001 | 0.79 (0.54–1.16) | 0.2263 |
| **mRS ≤2** | 0.54 (0.43–0.69) | <0.001 | 1.35 (0.79–2.30) | 0.2712 |
| **mRS ≤3** | 0.39 (0.30–0.51) | <0.001 | 0.69 (0.43–1.12) | 0.1297 |
| **On-admission treatment** |  |  |  |  |
| **tPA** | 1.92 (1.44–2.56) | <0.001 | 0.68 (0.48–0.96) | 0.0279 |
| **PCI** | 4.16 (1.75–9.89) | 0.0013 | 2.32 (0.86–6.28) | 0.0969 |
| **Thromboprophylaxis** | 3.57 (0.81–15.66) | 0.0921 | 1.77 (0.36–8.76) | 0.4834 |
| **Thrombectomy** | 3.63 (2.71–4.87) | <0.001 | 1.34 (0.94–1.91) | 0.1039 |
| **Severe impairment in consciousness (JCS ≥20)** | 12.18 (9.60–15.45) | <0.001 | 4.47 (3.41–5.87) | <0.001 |
| **Embolic infarction** | 3.87 (3.07–4.88) | <0.001 | 1.56 (1.16–2.09) | 0.0030 |
| **Comorbidity** |  |  |  |  |
| **Hypertension** | 0.58 (0.46–0.73) | <0.001 | 0.86 (0.66–1.11) | 0.2350 |
| **Diabetes** | 0.50 (0.36–0.71) | <0.001 | 0.68 (0.47–0.98) | 0.0394 |
| **Dyslipidemia** | 0.24 (0.16–0.36) | <0.001 | 0.52 (0.34–0.81) | 0.0040 |
| **Ischemic heart disease** | 0.91 (0.52–1.60) | 0.7497 |  |  |
| **Atrial fibrillation** | 2.68 (2.13–3.38) | <0.001 | 1.20 (0.91–1.59) | 0.2041 |
| **Pneumonia** | 4.77 (3.24–7.02) | <0.001 | 2.28 (1.48–3.50) | <0.001 |
| **COPD or asthma** | 1.07 (0.39–2.91) | 0.9009 |  |  |
| **CRD** | 2.00 (1.28–3.13) | 0.0025 | 2.29 (1.39–3.76) | 0.0011 |
| **Anemia** | 0.70 (0.22–2.23) | 0.5507 |  |  |
| **Cancer** | 1.51 (0.98–2.33) | 0.064 |  |  |
| **Annual hospital volume, case/year,**  **top quartile** | 0.95 (0.66–1.36) | 0.7669 |  |  |

ADL, activities of daily living; SCU, stroke care unit; ICU, intensive care unit; CI, confidence interval; BMI, body mass index; mRS, modified Rankin scale; tPA, tissue plasminogen activator; PCI, percutaneous catheter intervention; JCS, Japan Coma Scale; COPD, chronic obstructive pulmonary disease; CRD, chronic renal disease

Description of data: This is a table that reports the univariate and multivariate analyses results of factors associated with in-hospital mortality.
