# Supplementary material for: Effect of admission in the stroke care unit versus intensive care unit on in-hospital mortality in patients with acute ischemic stroke
Source: BMC Neurol. 2023 Nov 13;23:402. doi: 10.1186/s12883-023-03454-6 (PMC10641943; doi:10.1186/s12883-023-03454-6)
Supplement: Supplementary file 4 — Additional file 4. Stratified analysis of in-hospital mortality in the matched sample. [file 12883_2023_3454_MOESM4_ESM.docx]

**Additional file 4. Stratified analysis of in-hospital mortality in the matched sample**

| **Variable** | **No. of patients** | **SCU** | **ICU** | **OR (95% CI)** | **P-value for interaction** |
| --- | --- | --- | --- | --- | --- |
| **All** | 960 vs. 960 | 57 (5.9) | 76 (7.9) | 0.73 (0.51–1.05) |  |
| **Age, years** |  |  |  |  | 0.0267 |
| **≤80** | 571 vs. 571 | 24 (4.2) | 31 (5.4) | 0.76 (0.44–1.32) |  |
| **>80** | 388 vs. 388 | 24 (6.2) | 42 (10.8) | 0.54 (0.32–0.92) |  |
| **Sex** |  |  |  |  | 0.0601 |
| **male** | 546 vs. 546 | 32 (5.9) | 40 (7.3) | 0.79 (0.49–1.27) |  |
| **female** | 398 vs. 398 | 21 (5.3) | 31 (7.8) | 0.66 (0.37–1.17) |  |
| **Stroke type** |  |  |  |  | 0.3294 |
| **cardioembolic** | 501 vs. 501 | 37 (7.4) | 43 (8.6) | 0.84 (0.54–1.34) |  |
| **others** | 449 vs. 449 | 12 (2.7) | 26 (5.8) | 0.45 (0.22–0.90) |  |
| **Thrombectomy** |  |  |  |  | <0.001 |
| **yes** | 184 vs. 184 | 21 (11.4) | 10 (5.4) | 2.24 (1.02–4.90) |  |
| **no** | 773 vs. 773 | 43 (5.6) | 63 (8.2) | 0.66 (0.44–0.99) |  |
| **tPA** |  |  |  |  | 0.3351 |
| **yes** | 273 vs. 273 | 19 (7.0) | 18 (6.6) | 1.06 (0.54–2.07) |  |
| **no** | 659 vs. 659 | 40 (6.1) | 55 (8.3) | 0.70 (0.47–1.08) |  |
| **Atrial fibrillation** |  |  |  |  | 0.0334 |
| **yes** | 313 vs. 313 | 19 (6.1) | 31 (9.9) | 0.59 (0.32–1.06) |  |
| **no** | 636 vs. 636 | 33 (5.2) | 39 (6.1) | 0.84 (0.52–1.35) |  |
| **Hypertension** |  |  |  |  | 0.6376 |
| **yes** | 408 vs. 408 | 18 (4.4) | 27 (6.6) | 0.65 (0.35–1.20) |  |
| **no** | 537 vs. 537 | 39 (7.3) | 46 (8.6) | 0.84 (0.54–1.30) |  |
| **Diabetes mellitus** |  |  |  |  | 0.0824 |
| **yes** | 178 vs. 178 | 2 (1.1) | 7 (3.9) | 0.28 (0.06–1.36) |  |
| **no** | 775 vs. 775 | 50 (6.5) | 64 (8.3) | 0.76 (0.52–1.12) |  |

Data are shown as numbers or means (standard deviations).

SCU, stroke care unit; ICU, intensive care unit; OR: odds ratio; CI, confidence interval; tPA, tissue plasminogen activator

Description of data: This is a table that reports the in-hospital mortality of matched patients admitted in SCUs and ICUs.
