# Supplementary material for: Effect of admission in the stroke care unit versus intensive care unit on in-hospital mortality in patients with acute ischemic stroke
Source: BMC Neurol. 2023 Nov 13;23:402. doi: 10.1186/s12883-023-03454-6 (PMC10641943; doi:10.1186/s12883-023-03454-6)
Supplement: Supplementary file 5 — Additional file 5. In-hospital mortality based on stroke type. [file 12883_2023_3454_MOESM5_ESM.docx]

**Additional file 5. In-hospital mortality based on stroke type**

| **Variable** | **Before propensity score matching** | | | | | **After propensity score matching** | | | | |
| --- | --- | --- | --- | --- | --- | --- | --- | --- | --- | --- |
|  | **No. of patients** | **SCU** | **ICU** | **OR (95% CI)** | **P-value** | **No. of patients** | **SCU** | **ICU** | **OR (95% CI)** | **P-value** |
| **Atherothrombotic infarction** | 2656 vs. 345 | 33 (1.2) | 13 (3.8) | 0.32 (0.17–0.62) | 0.001 | 331 vs. 331 | 4 (1.2) | 11 (3.3) | 0.36 (0.11–1.13) | 0.117 |
| **Lacuna infarction** | 1,042 vs. 27 | 1 (0.1) | 0 (0.0) | - | 1.000 | 23 vs. 23 | 0(0.0) | 0(0.0) | - | - |

Data are presented as numbers (rates). SCU, stroke care unit; ICU, intensive care unit; OR: odds ratio; CI, confidence interval

Description of data: This is a table that reports the in-hospital mortality of patients admitted in SCUs and ICUs based on stroke type before and after propensity score matching.
